# A comparison of three ^67/68^Ga-labelled exendin-4 derivatives for β-cell imaging on the GLP-1 receptor: the influence of the conjugation site of NODAGA as chelator

**DOI:** 10.1186/s13550-014-0031-9

**Published:** 2014-06-22

**Authors:** Andreas Jodal, Brigitte Lankat-Buttgereit, Maarten Brom, Roger Schibli, Martin Béhé

**Affiliations:** 1 Center for Radiopharmaceutical Sciences ETH-PSI-USZ, Paul Scherrer Institute, OIPA/103, Villigen 5232, Switzerland; 2 Faculty of Medicine, Department of Gastroenterology, Endocrinology and Metabolism, University of Marburg, Marburg 35037, Germany; 3 Department of Radiology and Nuclear Medicine, Radboud University Medical Center, Nijmegen 6525, The Netherlands; 4 Department of Chemistry and Applied Biosciences, ETH Zurich, Zurich 8092, Switzerland

**Keywords:** Exendin-4, β-cell imaging, GLP-1 receptor, Insulinoma, PET, SPECT

## Abstract

**Background:**

Various diseases derive from pathologically altered β-cells. Their function can be increased, leading to hyperinsulinism, or decreased, resulting in diabetes. Non-invasive imaging of the β-cell-specific glucagon-like peptide receptor-1 (GLP-1R) would allow the assessment of both β-cell mass and derived tumours, potentially improving the diagnosis of various conditions. We tested three new ^67/68^Ga-labelled derivatives of exendin-4, an agonist of GLP-1R, *in vitro* and *in vivo*. We determined the influence of the chelator NODAGA conjugated to resident lysines either at positions 12 and 27 or the C-terminally attached lysine at position 40 on the binding and kinetics of the peptide.

**Methods:**

Binding and internalisation of ^67^Ga-labelled Ex4NOD12, Ex4NOD27 and Ex4NOD40 were tested on Chinese hamster lung (CHL) cells stably transfected to express the GLP-1 receptor (GLP-1R). *In vivo* biodistribution of ^68^Ga-labelled peptides was investigated in CD1 nu/nu mice with subcutaneous CHL-GLP-1R positive tumours; the specificity of the binding to GLP-1R was determined by pre-injecting excess peptide.

**Results:**

All peptides showed good *in vitro* binding affinities to GLP-1R in the range of 29 to 54 nM. ^67/68^Ga-Ex4NOD40 and ^67/68^Ga-Ex4NOD12 show excellent internalisation (>30%) and high specific uptake in GLP-1R positive tissue, but high activity was also found in the kidneys.

**Conclusions:**

We show that of the three peptides, Ga-Ex4NOD40 and Ga-Ex4NOD12 demonstrate the most favourable *in vitro* properties and *in vivo* binding to GLP-1R positive tissue. Therefore, we conclude that the lysines at positions 12 and 40 might preferentially be utilised for modifying exendin-4.

## Background

β-cells are located in the islets of Langerhans and are responsible for the biosynthesis and secretion of insulin and the regulation of blood glucose levels. A pathological dysfunction of the β-cells may lead to imbalanced blood glucose and can be the cause for several serious diseases. Inusulinomas are β-cell-derived tumours that secrete insulin and can cause hyperinsulinism, potentially leading to both hypoglycemia and neuroglycopenic symptoms [[1]]. Hyperinsulinism can also be caused by hyperplastic β-cells that excrete excessive amounts of insulin, such as those found in cases of nesidioblastosis and congenital hyperinsulinism. An exact localisation of the foci for both insulinoma and β-cell hyperplasia is necessary as surgical resection of the affected tissue is the best treatment option [[2]–[5]]. All of the currently available non-invasive imaging modalities, however, suffer from a low sensitivity, which is especially an issue for detecting small insulinoma [[6],[7]].

Another serious medical condition that arises from dysfunctional β-cells is diabetes. Type 1 diabetes is caused by an autoimmune reaction towards the β-cells leading to cell death while type 2 diabetes results from long-term elevated blood glucose levels induced by either apoptotic β-cells or due to insufficient insulin response caused by insulin receptor dysfunction [[8]]. So far, the diagnosis of diabetes is only possible *via* indirect means, e.g. determination of plasma glucose or HbA1c, which is far from optimal since these markers only show up after more than 80% of the islets are lost and the progression of the disease is irreversible [[9]]. A non-invasive means of determining β-cell mass might allow an earlier discovery of a decrease, which potentially could allow a timely intervention. Additionally, it would allow accurate assessment of transplanted β-cells as a potential treatment for diabetes, which is not possible so far.

Generally speaking, it can be stated that β-cell imaging has the potential for an improved diagnosis and disease management for a variety of medical conditions.

One potential target is the glucagon-like peptide receptor-1 (GLP-1R), which is highly expressed on β-cells. Exendin-4, an analogue of the metabolically unstable endogenous ligand glucagon-like peptide-1 (GLP-1), has been successfully used to image insulinoma in human patients [[10]]. A common modification site to introduce various moieties for different purposes to exendin-4 is the free amine of a C-terminally attached lysine or cysteine [[11]–[14]]. Exendin-4, though, possesses two naturally occurring lysines at positions 12 and 27, which might be used as modification sites as well. However, reports on how altering the respective position affects the properties of the peptide are conflicting. Several papers cite the importance of K12 and K27 for the binding to the receptor while other groups modified these positions without negative effects on the binding affinity [[15]–[17]]. A further risk of modifying the native lysine residues is a potential steric interference with the side chains of the neighbouring amino acids, which can lead to a distorted secondary structure. This poses an additional issue as the α-helical structure is an important factor for the positioning of the functional groups of the amino acids that are responsible for the binding to the receptor [[18]]. Our objective in this work is to explore potential modification possibilities of exendin-4 besides the well-established C-terminal alterations. This might open new possibilities combining new functional moieties and increase the variety of tracers.

As native insulinoma cell lines tend to produce insulin, which after inoculation in mice often leads to fatal hypoglycemia, we decided to use CHL cells that were transfected to overexpress the GLP-1 receptor in both *in vitro* and *in vivo* experiments.

Since the number of β-cells is low (only 1% to 2% of the pancreas mass are β-cells) and β-cell-derived tumours are quite small, a sensitive detection method is necessary [[5],[19]]. Positron emission tomography (PET) is ideal for this purpose as it offers a very high sensitivity and good spatial resolution. While ^64^Cu is a widely used PET nuclide, it emits β^−^ that can lead to an increased radiation burden for the patient [[20]]. ^68^Ga, a generator-based PET nuclide used in this work, does not have this drawback. It is widely available and useful for clinical use. In addition, it can be both quickly and efficiently incorporated into the chelator 1-(1,3-carboxypropyl)-1,4,7-triazacyclononane-4,7-diacetic acid (NODAGA), a chelator that can be used for labelling not only with ^67/68^Ga but also with of other radiometals like ^111^In and Al^18^F that are suitable for imaging. [[21]]. Using ^67^Ga as a γ-emitter might be adequate for single-photon emission computed tomography (SPECT) imaging; however, since it is currently not available in sufficiently high purity, it cannot be used to label our peptides with high-enough specific activity for *in vivo* experiments. Nevertheless, in this study, it was used as a substitute for ^68^Ga in *in vitro* experiments since it has a more convenient half-life of 3.3 days.

In this work, we investigate the influence of the site at which the chelator NODAGA is attached to exendin-4 on its binding affinity towards the GLP-1 receptor. Specifically, we have generated three ^67^Ga-labelled peptides (Ex4NOD12, Ex4NOD27 and Ex4NOD40) and measured their affinities towards GLP-1R expressed on the surface of Chinese hamster lung (CHL) cells. We have also assessed both their internalisation kinetics and their stability in human blood plasma. Additionally, we monitored the biodistribution of ^68^Ga-labelled peptides in CHL-GLP-1R-positive tumour-bearing CD1 nu/nu mice.

## Methods

### Radiolabelling of the exendin-4 derivatives

**Figure 1:**
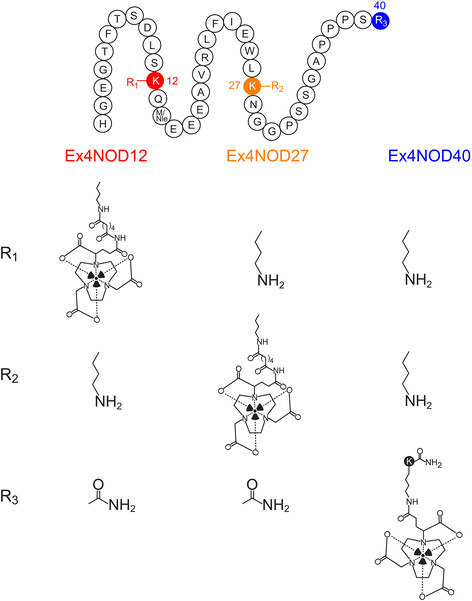
**Graphical representation of the three peptides.** Ex4NOD12 with the chelator attached to the lysine at position 12 (red), Ex4NOD27 with the chelator attached to the lysine at position 27 (yellow) and Ex4NOD40 with a C-terminally attached lysine at position 40 (blue). The other corresponding residues are listed in the table below.

^68^GaCl_3_ was eluted from a TiO_2_-based 1.85 GBq ^68^Ge/^68^Ga generator (Eckert & Ziegler, Berlin, Germany) with 0.1 N Ultrapure HCl (Merck Millipore, Switzerland).

Ex4NOD12, Ex4NOD27 and Ex4NOD40, shown in Figure 1, were synthetised by Peptide Specialty Laboratories (Heidelberg, Germany). [Lys^40^(Ahx-DTPA)NH_2_]-exendin-4 (HGEGTFTSDLSKQMEEEAVRLFIEWLKNGGPSSGAPPPSK(DTPA-Ahx)-NH_2_) and [Lys^40^(DTPA)]exendin-3 (HSDGTFTSDLSKQMEEEAVRLFIEWLKNGGPSSGAPPPSK(DTPA)-NH_2_), synthetised by Peptide Specialty Laboratories were used as already established standards. The chelators NODAGA and diethylene triamine pentaacetic acid (DTPA) were conjugated to the ε-amino group of the lysine either at position 12 or 27 with aminocaproic acid (Ahx) as spacer or attached to a lysine, which was linked to the C-terminal end of the peptide leading to a lysine in position 40. In either case, the C-terminal carboxyl group was amidated. In order to improve oxidative stability, the methionine in position 14 of Ex4NOD40 was replaced with norleucine in both Ex4NOD12 and Ex4NOD27.

The generator was eluted drop by drop, fractions of 200 μL each were collected, and the fractions with the highest activity were used for labelling.

Labelling of all peptides was performed in 4-(2-hydroxyethyl)-1-piperazineethanesulphonic acid (HEPES) buffer in a final concentration of 0.25 M and pH 4.5. 0.23 to 0.41 nmol peptide was added to 60 to 77 MBq ^68^GaCl_3,_ followed by a 15-min incubation of the reaction mixture at 90°C. For quality control, the sample was diluted in 0.1 mM sodium DTPA and analysed using reversed-phase high-performance liquid chromatography (RP-HPLC) on a C18 reversed-phase column (Dr. Maisch Reprospher 300 C18-TN, 46 mm × 150 mm; 5 μm). The column was eluted with water containing 0.1% trifluoroacetic acid (TFA), with a linear gradient from 15% to 95% acetonitrile for 10 min followed by an isocratic elution at 95% acetonitrile for additional 5 min with a flow rate of 1 mL/min.

^67^Ga labellings were performed using 55 to 65 MBq ^67^GaCl_3_ (Mallinckrodt, Netherlands) and 1 to 6.75 nmol Ex4NOD40, Ex4NOD12 and Ex4NOD27 in 10 μL 0.4 M ammonium acetate pH 5.5 and 20 μL Milli-Q water followed by 30 min incubation at 90°C. Quality control was performed as described above.

### Cell culture

Chinese hamster lung cell line stably transfected with the GLP-1 receptor gene (CHL-GLP-1R positive cells) were cultured in Dulbecco's modified Eagle's medium (DMEM) with 4.5 g/L d-glucose and GlutaMax. In addition, the media contained 10% fetal calf serum, 100 IU/mL penicillin G, 10 mg/mL streptomycine, 500 μg/mL geneticin sulfate, 1 mM sodium pyruvate and 0.1 mM non-essential amino acids. The cells were maintained in a humidified 5% CO_2_ atmosphere at 37°C and were harvested by trypsinisation with trypsin/EDTA.

### Labelling of exendin-4 derivatives with stable isotopes

All peptides were labelled by adding 40 μL of the respective 0.25 mM peptide solution and 2 μL of a 10 mM ^nat^GaCl_3_ (Sigma-Aldrich, Switzerland) solution in 60 μL 0.4 M ammonium acetate buffer (pH 5.5) followed by a 15-min incubation at 90°C. The labelling was verified by liquid chromatography-mass spectrometry (LC/MS) on an Atlantis C18 (25 cm × 4.6 mm; 5 μm) column.

### IC_50_ determination

The half-maximal inhibitory concentrations (IC_50_) of ^nat^Ga-labelled Ex4NOD12, Ex4NOD27 and Ex4NOD40 were determined using CHL-GLP-1R positive cells grown on six well plates (approximately 0.8 × 10^6^ cells/well grown overnight). The two already published peptides ^nat^In-labelled [Lys^40^(DTPA)]exendin-3 and [Lys^40^(Ahx-DTPA)NH_2_]-exendin-4 were used to determine if the cell line has an influence on the results. A 4-kBq (120 fmol)-^67^Ga-labelled Ex4NOD40 was used for detection of the binding. The cells were washed twice with PBS and incubated for 60 min on ice with 100 μL (4 kBq; 120 fmol) ^67^Ga-labelled Ex4NOD40 in the presence of increasing concentrations of non-radioactive-labelled exendin-4 derivative (10^−11^ to 10^−6^ M). The total volume was adjusted with media (DMEM with 0.1% BSA) to 1 mL. For the total binding, no ^nat^Ga-labelled peptide was added. After incubation with the labelled peptides, the cells were washed twice with phosphate buffered saline (PBS), solubilised with 1 mL sodium hydroxide (NaOH) and collected and the activity was quantified using a γ-counter (Packard Cobra II Auto Gamma, PerkinElmer, Switzerland). The IC_50_ values were calculated by fitting the data with non-linear regression using least squares fit with GraphPad Prism (GraphPad Software, La Jolla, CA, USA). Experiments were performed on triplicate samples.

### Internalisation assay

The internalisation kinetics of ^67^Ga-Ex4NOD12, ^67^Ga-Ex4NOD27 and ^67^Ga-Ex4NOD40 were determined using CHL-GLP-1 receptor positive cells as described above. A 4 kBq (120 fmol) of the respective ^67^Ga-labelled peptide was used as a probe. Cells were incubated for specific time points (5, 15, 30, 60 and 120 min, respectively) at 37°C; non-specific binding was determined by adding an additional 100 μL of ^nat^Ga-labelled tracer to a final concentration of 1 μM of the corresponding peptide. After incubation, the supernatant was aspirated and the wells were washed with 1 mL PBS. Both the supernatant and wash fractions were pooled and used to determine the non-bound fraction. In order to dislodge the surface-bound peptide, the cells were incubated at room temperature with 1 mL glycine buffer pH 2.6 for 5 min. The glycine wash was collected separately. The internalised fraction was identified by adding 1 mL 1 M NaOH to the cells with subsequent collection of the lysates. The activity in all three fractions was measured in a γ-counter (Packard Cobra II Auto Gamma, Perkin Elmer, Switzerland). Experiments were performed on triplicate samples.

### Plasma stability

A 5-MBq (0.2 nmol)-labelled peptide was added into 250 μL fresh human blood plasma and incubated at 37°C for 48 h. Forty-microlitre samples were taken at specific time points (0, 1, 4, 24, and 48 h) and mixed with 200 μL precipitation solution (methanol/water, 0.1% TFA 1:1). The sample was then filtered through a Thomson Single StEP Filter vial 0.45 μm PVDF (Thomson Instrument Company, Oceanside, CA, USA) and analysed *via* RP-HPLC on a Discovery BioWide Pore C18 (15 cm × 2.1 mm; 3 μm) column. The column was eluted with water containing 0.1% TFA, with a linear gradient from 15% to 95% acetonitrile for 10 min followed by an isocratic elution at 95% acetonitrile for an additional 5 min with a flow rate of 1 mL/min.

### Biodistribution

All *in vivo* experiments were approved by the local veterinarian department and conducted in accordance with the Swiss law of animal protection. Biodistribution studies of ^68^Ga-Ex4NOD12, ^68^Ga-Ex4NOD27 and ^68^Ga-Ex4NOD40 were compared. Six-week-old female CD1 nu/nu mice were subcutaneously inoculated in both shoulder regions with 8 × 10^6^ CHL-GLP-1R positive cells suspended in PBS pH 7.4. After 4 weeks, the mice were randomly divided into groups of four mice each. The mice were injected with approximately 750 kBq (3 to 8 pmol) of the respective ^68^Ga-labelled peptide *via* the tail vein. The mice were sacrificed at specific time points (0.5, 1, 2 h). In order to determine GLP-1 receptor-mediated uptake, one group per peptide was co-injected with excess (100 μg each) unlabelled peptide and euthanised 2 h after injection. Blood, heart, lungs, spleen, kidneys, pancreas, stomach, intestine, liver, muscle, bone and tumour were removed and weighed and activity was determined in a γ-counter (Packard Cobra II Auto Gamma, Perkin Elmer, Switzerland). The percentage injected activity per gram tissue (%iA/g) was calculated for each tissue. The statistical significance was determined using Student's unpaired *t* test.

### Circular dichroism

Three sets of spectra were recorded for each peptide with a concentration of 20 μM in Milli-Q water using a Chirascan spectropolarimeter (Applied Photophysics, UK) with a 0.1-cm path length in the range of 190 to 280 nm. First, a baseline CD spectrum was recorded at 20°C followed by thermal denaturation at 94°C for 30 min. After the denaturation, a spectrum was recorded to determine the linearisation of the peptide. Subsequently, the sample was cooled to 20°C and another set of spectra was recorded to evaluate the refolding of the peptide.

## Results

### Radiolabelling

All peptides were labelled with ^67^Ga with a specific activity between 5.6 to 35 MBq/nmol. The specific activity achieved for ^68^Ga labelling ranged from 71 to 163 MBq/nmol at the time of injection. Radiochemical purity was >95% as determined by HPLC. ^67/68^Ga-DTPA eluted from the column after 2 min whereas ^67/68^Ga-labelled Ex4NOD12, Ex4NOD27 and Ex4NOD40 had a retention time of approximately 8 min.

### Circular dichroism

CD spectroscopy showed partial unfolding of all peptides after 30 min at 95°C. After subsequent cooling to 20°C, spectra were identical to the ones taken before thermal denaturation. This indicates a complete refolding of the peptides into their natural α-helical structure.

### IC_50_ binding assay

**Figure 2:**
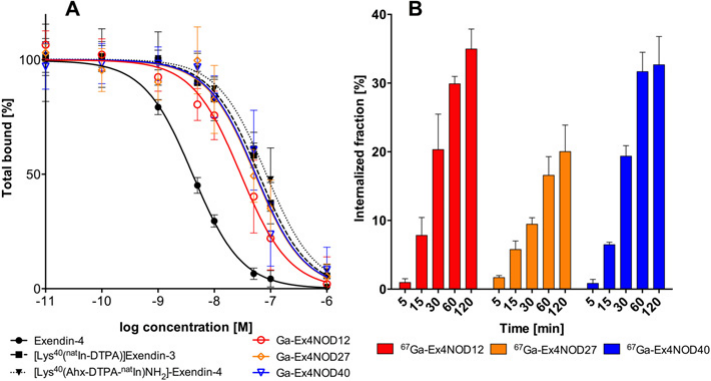
**Competition binding assay (A) and internalisation kinetics (B).(A)** IC 50 values of ^nat^Ga-Ex4NOD12, ^nat^Ga-Ex4NOD27, ^nat^Ga-Ex4NOD40, exendin-4, [Lys^40^(^nat^In-DTPA)]exendin-3 and [Lys^40^(Ahx-DTPA-^nat^In)NH_2_]-exendin-4 in CHL-GLP-1R positive cells. A 120-fmol ^67^Ga-Ex4NOD40 with a specific activity of 8.9 MBq/nmol was used as tracer. **(B)** Internalisation of ^67^Ga-Ex4NOD12, ^67^Ga-Ex4NOD27 and ^67^Ga-Ex4NOD40 in CHL-GLP-1R positive cells. A 120-fmol ^67^Ga-labelled peptide was used as tracer. The values are corrected for non-specific binding determined by co-incubation with 1 μM ^nat^Ga-labelled peptide.

The results of the IC_50_ determination are shown in Figure 2A. The IC_50_ for ^nat^Ga-Ex4NOD12, ^nat^Ga-Ex4NOD27 and ^nat^Ga-Ex4NOD40 for binding to CHL-GLP-1R positive cells were 29 nM (95% confidence interval 22 to 40 nM), 53 nM (95% confidence interval 38 to 73 nM) and 54 nM (95% confidence interval 39 to 76 nM), respectively. The IC_50_ of non-modified exendin-4 was 4.1 nM (95% confidence interval 3.6 to 4.7 nM). [Lys^40^(^nat^In-DTPA)]exendin-3 and [Lys^40^(Ahx-DTPA-^nat^In)NH_2_]-exendin-4, previously tested on INS-1 cells, showed an IC_50_ of 62 nM (95% confidence interval 51 to 75 nM) and 89 nM (95% confidence interval 66 to 120 nM) on the cell line used in these experiments.

### Internalisation assay

Figure 2B summarises the internalisation kinetics of ^67^Ga-Ex4NOD40, ^67^Ga-Ex4NOD12 and ^67^Ga-Ex4NOD27 in CHL-GLP-1R-positive cells over the course of 2 h. ^67^Ga-Ex4NOD12 and ^67^Ga-Ex4NOD40 show a similar internalisation of 32.7% ± 4.1% and 35.0% ± 2.9%, respectively after 120 min. Ex4NOD27 shows both a slower and significantly lower (*p* < 0.02) internalisation of 20.0% ± 3.9% after 120 min.

### Plasma stability

**Figure 3:**
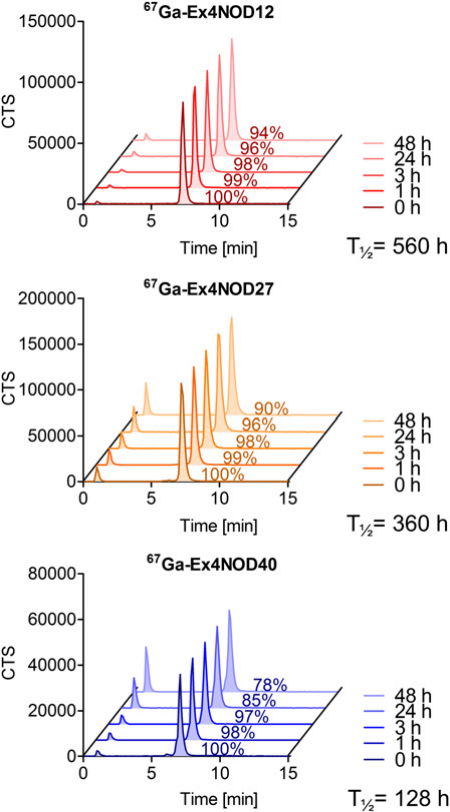
**Plasma stability of**^**67**^**Ga-Ex4NOD12,**^**67**^**Ga-Ex4NOD27 and**^**67**^**Ga-Ex4NOD40 in fresh human blood plasma.** The experiment was performed over the course of 48 h.

Figure 3 illustrates the plasma stability in fresh human blood plasma. Seventy-eight percent of ^67^Ga-Ex4NOD40, 94% of ^67^Ga-Ex4NOD12 and 90% of ^67^Ga-Ex4NOD27 were intact after 48 h. The half-lives were 128, 560 and 360 h, respectively.

### Biodistribution

**Figure 4:**
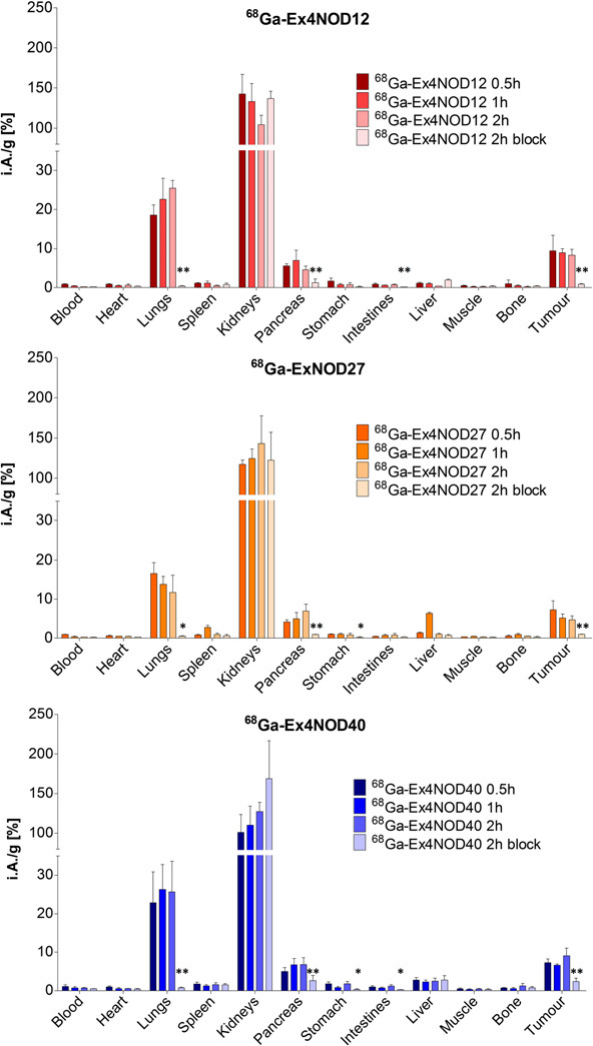
**Biodistribution of**^**68**^**Ga-Ex4NOD12,**^**68**^**Ga-Ex4NOD27 and**^**68**^**Ga-Ex4NOD40 in CD1 nu/nu mice with subcutaneous CHL-GLP-1R-positive tumours.** Values are mean percentages injected dose per gram tissue (*n* = 4. Error bars SD). Blocking was performed by pre-injection of 100 μg excess of the corresponding unlabelled compound. Mice were sacrificed 0.5, 1 and 2 h after injection. The significance between the 2 h unblocked and blocked was tested by Student's unpaired *t* test (*p < 0.05; **p < 0.01).

Figure 4 and Table 1 show the biodistribution of ^68^Ga-Ex4NOD40, ^68^Ga-Ex4NOD12 and ^68^Ga-Ex4NOD27 after 2 h as well as the blocking experiment with additionally pre-injected excess peptide. All compounds showed a high specific tumour uptake as well as specific uptake in the pancreas and lung. Generally, ^68^Ga-labelled Ex4NOD12 and Ex4NOD40 demonstrated very similar uptake; ^68^Ga-Ex4NOD27, however, displayed a significantly lower accumulation in the lung (*p* < 0.05) and, at some time points, in the tumour (*p* < 0.05). The renal uptake of all the ^68^Ga-labelled probes was high and excess unlabelled peptide did not block the kidney uptake indicating that it is not GLP-1 receptor mediated which is in line with earlier observations [[22]].

**Table 1:** **Biodistribution of the**^**68**^**Ga-labelled peptides in CD1 nu/nu mice**

	**Biodistribution (%iA/g)**			
	**0.5 h**	**1 h**	**2 h**	**2 h blocked**
**Ex4NOD12**				
Blood	0.84 ± 0.11	0.32 ± 0.16	0.18 ± 0.01	0.19 ± 0.04
Heart	0.78 ± 0.13	0.44 ± 0.07	0.51 ± 0.35	0.26 ± 0.06
Lung	18.59 ± 2.62	22.67 ± 5.36	25.47 ± 2.02	0.37 ± 0.08
Spleen	1.06 ± 0.11	1.06 ± 0.56	0.44 ± 0.05	0.75 ± 0.31
Kidneys	142.52 ± 24.51	133.35 ± 22.18	104.20 ± 12.02	136.72 ± 9.41
Pancreas	5.57 ± 0.53	6.93 ± 2.64	4.52 ± 0.96	1.23 ± 0.91
Stomach	1.59 ± 0.78	0.77 ± 0.24	0.75 ± 0.49	0.20 ± 0.07
Intestines	0.83 ± 0.23	0.52 ± 0.05	0.74 ± 0.10	0.13 ± 0.02
Liver	1.09 ± 0.19	0.91 ± 0.19	0.31 ± 0.05	1.88 ± 0.23
Muscle	0.43 ± 0.11	0.21 ± 0.03	0.19 ± 0.07	0.26 ± 0.16
Bone	0.98 ± 0.90	0.42 ± 0.16	0.23 ± 0.02	0.36 ± 0.10
Tumour	9.44 ± 3.94	8.92 ± 1.08	8.29 ± 1.50	0.83 ± 0.17
**Ex4NOD27**				
Blood	0.88 ± 0.09	0.36 ± 0.09	0.24 ± 0.04	0.23 ± 0.05
Heart	0.62 ± 0.05	0.44 ± 0.02	0.41 ± 0.04	0.27 ± 0.02
Lung	16.56 ± 2.74	13.79 ± 2.04	11.63 ± 4.51	0.43 ± 0.18
Spleen	0.81 ± 0.08	2.72 ± 0.55	0.89 ± 0.40	0.58 ± 0.30
Kidneys	117.21 ± 5.47	124.50 ± 12.13	143.00 ± 34.71	122.30 ± 35.13
Pancreas	4.10 ± 0.54	4.91 ± 1.61	6.89 ± 1.81	0.87 ± 0.10
Stomach	0.93 ± 0.16	0.92 ± 0.27	0.79 ± 0.39	0.19 ± 0.13
Intestines	0.40 ± 0.07	0.66 ± 0.19	0.71 ± 0.40	0.24 ± 0.11
Liver	1.36 ± 0.10	6.31 ± 0.27	1.04 ± 0.17	0.65 ± 0.22
Muscle	0.33 ± 0.04	0.38 ± 0.09	0.29 ± 0.05	0.18 ± 0.10
Bone	0.56 ± 0.16	0.89 ± 0.22	0.47 ± 0.08	0.29 ± 0.11
Tumour	7.26 ± 2.32	5.18 ± 0.98	4.69 ± 0.98	0.97 ± 0.07
**Ex4NOD40**				
Blood	1.15 ± 0.37	0.72 ± 0.33	0.66 ± 0.17	0.48 ± 0.10
Heart	0.94 ± 0.34	0.56 ± 0.24	0.57 ± 0.08	0.41 ± 0.12
Lung	22.83 ± 8.04	26.28 ± 6.46	25.67 ± 7.94	0.77 ± 0.12
Spleen	1.73 ± 0.51	1.23 ± 0.37	1.60 ± 0.43	1.50 ± 0.38
Kidneys	101.14 ± 22.60	110.35 ± 24.27	127.51 ± 11.58	168.66 ± 48.22
Pancreas	5.05 ± 1.02	6.73 ± 1.65	6.80 ± 1.80	2.63 ± 1.34
Stomach	1.80 ± 0.48	0.82 ± 0.28	1.78 ± 0.67	0.34 ± 0.12
Intestines	1.01 ± 0.34	0.72 ± 0.17	1.19 ± 0.37	0.31 ± 0.03
Liver	2.71 ± 0.71	2.28 ± 0.44	2.47 ± 0.77	2.75 ± 1.13
Muscle	0.46 ± 0.20	0.34 ± 0.16	0.44 ± 0.17	0.33 ± 0.14
Bone	0.66 ± 0.16	0.58 ± 0.22	1.24 ± 0.67	0.75 ± 0.35
Tumour	7.30 ± 0.94	6.60 ± 0.35	9.04 ± 1.97	2.39 ± 0.87

The position of the chelator appears to affect the *in vivo* kinetics of the peptide. Both ^68^Ga-Ex4NOD12 and ^68^Ga-Ex4NOD40 show a high uptake in the lung that is increasing over the course of 2 h. ^68^Ga-Ex4NOD27 in contrast exhibits a lower uptake which in addition decreases with time. Kidney uptake seems to be affected as well, as ^68^Ga-Ex4NOD12 shows a reduction of activity over time while the other two radiolabelled peptides seem to accumulate. Additionally, ^68^Ga-Ex4NOD12 shows the lowest non-specific uptake tissue not expressing GLP-1R as well as in the blocking experiments (tumour *p* < 0.04, lung *p* < 0.003). For the pancreas, however, the uptake peaks at 1 h, while the other two probes show an increasing uptake for the time of the experiment.

## Discussion

The GLP-1 receptor is specifically expressed on β-cells and is a promising target for β-cell imaging. Radiolabelled derivatives of exendin-4, a ligand for GLP-1R, have been successfully used to image insulinoma in patients [[10]]. There are conflicting reports as to whether the conjugation site of the chelator affects the properties of the peptides. Kirkpatrick et al. showed that both lysine residues at positions 12 and 27 are important for the affinity to the GLP-1 receptor as they form salt bridges that stabilise the binding [[15]]. However, experiments by Son et al. revealed that conjugation of bile acid derivatives to either K12 or K27 still result in a sub-nanomolar affinity of the peptide. In contrast, the conjugation at both positions leads to a loss of affinity of a factor of at least 30, depending on the substrate [[23]]. Jin et al. were also able to show that biotinylated exendin-4 derivatives showed no significantly lower affinities compared to unmodified exendin-4 when conjugated at the same positions [[17]]. In contrast, the investigations of Kim et al. on PEGylated exendin-4 derivatives led to the conclusion that conjugations at both K27 and a C-terminally added cysteine had similar affinities as exendin-4, while the K12 derivative showed reduced binding to the receptor [[16]].

In order to elucidate the effects of modifications on our construct, we compared three ^67/68^Ga-labelled peptides Ex4NOD12, Ex4NOD27 and Ex4NOD40 with different conjugation positions of the chelator NODAGA, which allows the incorporation of a variety of radio metals. CD measurement shows the formation of an α-helix for all three peptides similar to the unmodified exendin-4. In addition, all peptides exhibit complete refolding to its original α-helical structure after denaturation at 94°C, the temperature used for labelling. IC_50_-binding studies on transfected CHL cells stably expressing the GLP-1 receptor revealed that all modified peptides showed a 7- to 13-fold lower binding compared to the unmodified exendin-4. Ga-Ex4NOD12 showed the highest affinity to the receptor whereas both Ga-Ex4NOD40 and Ga-Ex4NOD27 showed to be similar, albeit lower binding affinities. This is in contrast to reports in which C-terminally modified exendin-4 derivatives show similar binding as the unmodified peptide [[11],[16],[24]]. The differences to literature values can be attributed to the fact that different cell lines were used. The results show a 5- and 42-fold lower binding affinity compared to the published data obtained on INS-1 cells, which indicates an overall lower affinity of modified exendin derivatives to the GLP-1 receptor in this cell line [[24],[25]].

All peptides show a high metabolic stability in fresh human blood plasma for up to 3 h. As imaging with ^68^Ga is usually performed within 1 to 2 h after injection, the stability of the peptides is sufficient. Ga-Ex4NOD12 and Ga-Ex4NOD27 even show an improved stability over Ga-Ex4NOD40 for more than 48 h. The bulky chelator might influence the accessibility of intramolecular cleavage sites of enzymes leading to a lowered metabolism.

Biodistribution in CD1 nu/nu mice with a subcutaneous CHL-GLP-1-receptor-positive tumour showed specific uptake in tumour and GLP-1-receptor-positive tissue as well as high non-GLP-1R-mediated kidney absorption for all three peptides. However, both the amount and kinetics of the accumulated activity differ. Retained activity in GLP-1R-positive tissue in ^68^Ga-Ex4NOD12- and ^68^Ga-Ex4NOD40-injected mice are similar, while that in ^68^Ga-Ex4NOD27-injected mice shows lower accumulation in some time points in the tumour and the lung. As Ga-Ex4NOD40 and Ga-Ex4NOD27 have similar IC_50_ values, comparable uptake would be expected. Subsequent internalisation experiments, however, revealed that internalisation of Ga-Ex4NOD27 is significantly lower than the other two peptides.

One additional challenge when imaging β-cells is the limited amount of overall GLP-1 receptors available which requires the administration of low amounts of peptide with a high specific activity in order to avoid receptor blocking [[13]]. One potential way to further increase the specific activity and therefore reduce the amount of tracer necessary to obtain a clear image is to attach several chelators to the peptide allowing more radioactivity per molecule. Another potential advantage of two conjugation sites might be the attachment of multiple moieties to exendin-4, for example a fluorescent dye and a chelator, which could be used as a probe for dual imaging. These experiments show that conjugating NODAGA to either the lysines in position 12 or 40 and to a lesser extent in position 27, does not greatly influence the properties of the peptide and could be used as potential attachment sites for various moieties. Brom et al. have shown comparable results using the ^68^Ga-labelled exendin derivative [Lys^40^(^68^Ga-DOTA)]exendin-3. Biodistribution in Balb/c nude mice showed similar uptake in the pancreas (6.7 ± 1.8%iA/g), INS-1 tumour (8.9 ± 3.1%iA/g) and kidneys 1 h after injection [[12]]. ^68^Ga-Ex4NOD40 showed a slightly higher pancreas uptake, whereas the kidney and tumour uptake were lower compared to [Lys^40^(^68^Ga-DOTA)]exendin-3. For Ex4NOD12, the overall uptake in the pancreas, tumour and kidney was higher, and for Ex4NOD27, the pancreas and tumour uptake were lower, while the kidney uptake was the same. Wild et al. performed experiments with [Lys^40^ (Ahx-DOTA-^68^Ga)NH_2_]-exendin-4 in Rip1Tag2 mice with spontaneously occurring insulinoma tumours [[26]]. As spontaneous and subcutaneous tumours are inherently different, a direct comparison of tumour-to-normal organ ratios between Wild's and our work is difficult. Therefore, we compared uptake of our tracer in GLP-1R-positive (pancreas) and GLP-1R-negative tissue (muscle and kidneys) with data from Wild et al. The pancreas-to-muscle ratio after of [Lys^40^ (Ahx-DOTA-^68^Ga)NH_2_]-exendin-4 is lower compared to the peptides investigated in this paper, while the pancreas-to-kidney ratios are comparable or slightly higher, indicating a good target to background contrast for the three tested peptides.

## Conclusions

In conclusion, ^68^Ga-Ex4NOD12, ^68^Ga-Ex4NOD40 and, to a lesser extent, ^68^Ga-Ex4NOD27 show comparable results and demonstrate good properties for pancreatic β-cell imaging, as they show high uptake and retention in GLP-1R-positive tumour and tissue. ^68^Ga as a radionuclide allows fast and high specific labelling, with a half-life matching the biological half-life of the peptide [[27]]. Our results show that the lysines at positions 12 and 40 could be preferentially used to modify exendin-4.

## Abbreviations

%iA/g: percentage injected activity per gram tissue: 

CD: circular dichroism: 

CHL: Chinese hamster lung: 

DMEM: Dulbecco's modified Eagle's medium: 

DTPA: diethylene triamine pentaacetic acid: 

Ex4NOD12: HGEGTFTSDLSK(NODAGA-Ahx)QNleEEEAVRLFIEWLKNGGPSSGAPPPS-NH_2_: 

Ex4NOD27: HGEGTFTSDLSKQNleEEEAVRLFIEWLK(NODAGA-Ahx)NGGPSSGAPPPS-NH_2_: 

Ex4NOD40: HGEGTFTSDLSKQMEEEAVRLFIEWLKNGGPSSGAPPPSK(NODAGA)-NH_2_: 

GLP-1: glucagon-like peptide: 

GLP-1R: glucagon-like peptide receptor-1: 

HbA1c: % glycated hemoglobin in blood: 

HEPES: 4-(2-hydroxyethyl)-1-piperazineethanesulphonic acid: 

IC_50_: half-maximal inhibitory concentrations: 

LC/MS: liquid chromatography-mass spectrometry: 

[Lys^40^(Ahx-DTPA)NH_2_]-exendin-4: HGEGTFTSDLSKQMEEEAVRLFIEWLKNGGPSSGAPPPSK(DTPA-Ahx)-NH_2_: 

[Lys^40^(DTPA)]exendin-3: HSDGTFTSDLSKQMEEEAVRLFIEWLKNGGPSSGAPPPSK(DTPA)-NH_2_: 

NaOH: sodium hydroxide: 

NODAGA: 1-(1,3-carboxypropyl)-1,4,7- triazacyclononane-4,7-diacetic acid: 

PBS: phosphate buffered saline: 

PET: positron emission tomography: 

RP-HPLC: reversed-phase high-performance liquid chromatography: 

SPECT: single-photon emission computed tomography: 

TFA: trifluoroacetic acid: 

## Competing interests

Patent disclosure: Martin Béhé declares that he is an inventor of the patent: Invention affecting GLP-1 and exendin. Philipps-Universität Marburg, June 17th 2009. All other authors declare that they have no conflict of interest.

## Authors' contributions

AJ carried out the experiments and wrote the manuscript. BLB was involved in the experiments and the analysis of the transfected cells and revised the manuscript. MBr revised the manuscript. RS was involved in the design and analysis of the experiments and revised the manuscript. MBe designed the studies and analysed the results as well as wrote parts of the manuscript. All authors read and approved the manuscript.
